# Recognition of Frequency Modulated Whistle-Like Sounds by a Bottlenose Dolphin (*Tursiops truncatus*) and Humans with Transformations in Amplitude, Duration and Frequency

**DOI:** 10.1371/journal.pone.0147512

**Published:** 2016-02-10

**Authors:** Brian K. Branstetter, Caroline M. DeLong, Brandon Dziedzic, Amy Black, Kimberly Bakhtiari

**Affiliations:** 1 National Marine Mammal Foundation, San Diego, California, United States of America; 2 Department of Psychology, College of Liberal Arts, Rochester Institute of Technology, Rochester, New York, United States of America; University of St Andrews, UNITED KINGDOM

## Abstract

Bottlenose dolphins (*Tursiops truncatus*) use the frequency contour of whistles produced by conspecifics for individual recognition. Here we tested a bottlenose dolphin’s (*Tursiops truncatus*) ability to recognize frequency modulated whistle-like sounds using a three alternative matching-to-sample paradigm. The dolphin was first trained to select a specific object (object A) in response to a specific sound (sound A) for a total of three object-sound associations. The sounds were then transformed by amplitude, duration, or frequency transposition while still preserving the frequency contour of each sound. For comparison purposes, 30 human participants completed an identical task with the same sounds, objects, and training procedure. The dolphin’s ability to correctly match objects to sounds was robust to changes in amplitude with only a minor decrement in performance for short durations. The dolphin failed to recognize sounds that were frequency transposed by plus or minus ½ octaves. Human participants demonstrated robust recognition with all acoustic transformations. The results indicate that this dolphin’s acoustic recognition of whistle-like sounds was constrained by absolute pitch. Unlike human speech, which varies considerably in average frequency, signature whistles are relatively stable in frequency, which may have selected for a whistle recognition system invariant to frequency transposition.

## Introduction

Whistle use by bottlenose dolphins serves several functions including broadcasting individual identification [1–3], a vocal label to address individual conspecifics [4], maintaining group cohesion [5, 6], long range communication [7, 8], recruitment during feeding [9] and advertising emotional state [10, 11]. One whistle type that has been the focus of much study is the “signature whistle” [2]. Signature whistles can be defined as “a learned, individually distinctive whistle type in a dolphin’s repertoire that broadcasts the identity of the whistler” [12]. Use of a unique vocalization to identify conspecifics can be important in the visually restricted environment of the ocean. In bottlenose dolphins, signature whistles can account for approximately 80–100% of all whistles when bottlenose dolphins are isolated [13] and approximately 30–70% of whistles for free swimming dolphins [12]. Playback studies have demonstrated that wild dolphins are more likely to orient towards an underwater speaker projecting a whistle from a more familiar dolphin compared to a whistle from a less familiar dolphin [3]. This differential response is often interpreted as evidence of recognition or that the animal has learned to associate the whistle with the whistler. However, an argument could also be made that the dolphins’ differential behavior is simply two different responses to familiar vs. unfamiliar whistles, without any direct knowledge of the whistler’s identity. Unequivocal proof that dolphins recognize the referential component of a signature whistle requires direct evidence of a learned association between a whistle and the specific animal that produced the whistle. In the laboratory, dolphins have demonstrated the ability to understand the referential component of acoustic (and visual) symbols by learning to associate specific sounds with specific objects or actions [14, 15]. Dolphin cognitive abilities demonstrated in the laboratory along with observations and experiments conducted in the field provide compelling evidence that wild dolphins likely recognize each other based on signature whistles.

**Fig 1:**
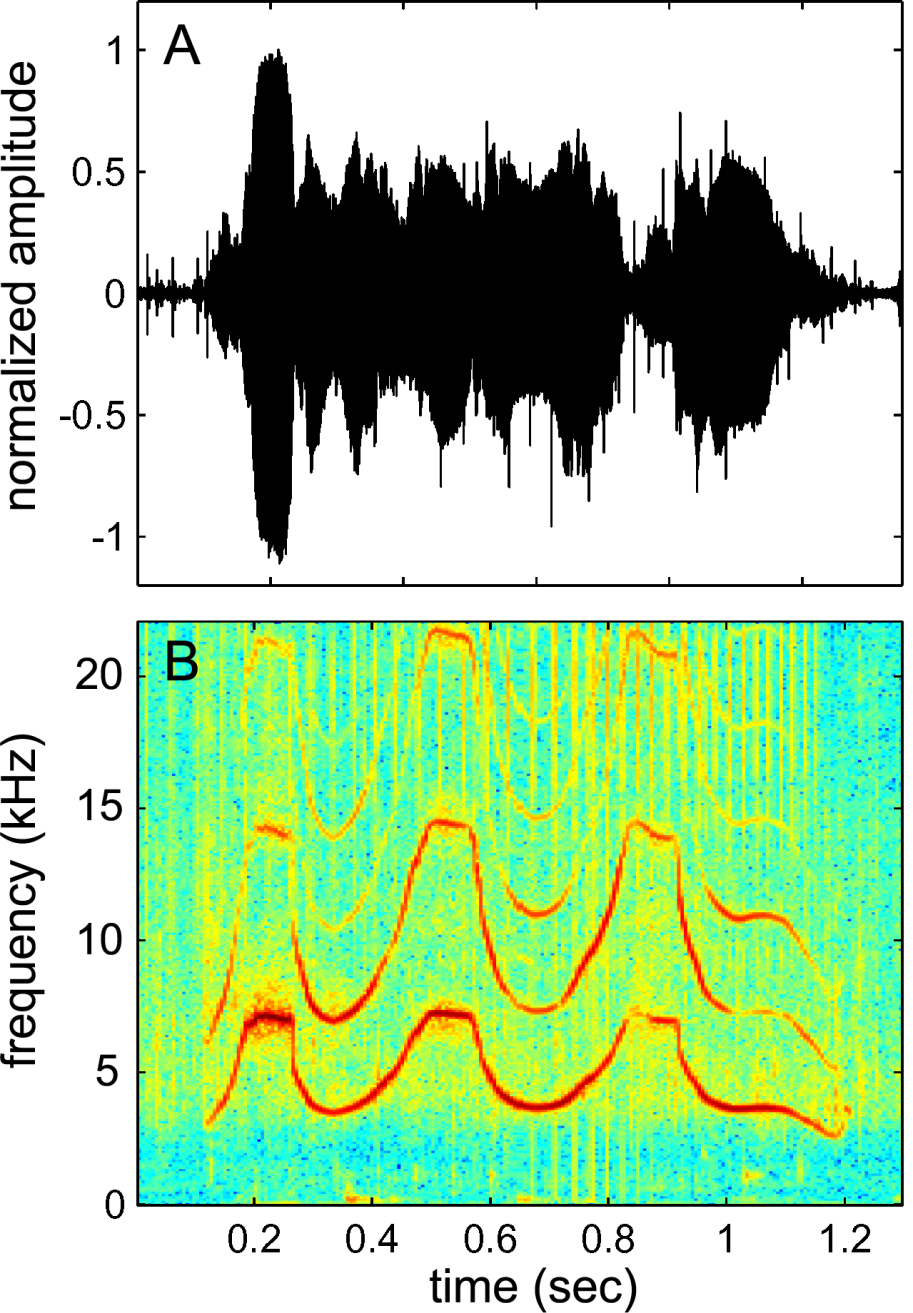
Signature whistle of the dolphin SAY. (A) Waveform of the whistle and (B) spectrogram of the same whistle displaying the frequency contour that dolphins use for recognition. The whistle contains several harmonics.

Dolphin whistles can vary in amplitude and duration, have repeating loops, and contain “voice” features [16]. However, the stereotyped frequency contour (i.e., a graph of whistle frequency over time) of the whistle appears to contain the information used for recognition (see Fig 1) [16]. Whistle recognition is further complicated by the fact that the frequency contour of a signature whistle can vary in many acoustic parameters (e.g., number of loops, start frequency, duration, frequency of inflection points, amplitude, etc) while still maintaining the relational aspects of the frequency contour pattern [11, 17]. These observations suggest the dolphin’s ability to recognize whistles must be flexible to some acoustic transformations. Laboratory evidence supports this assumption. For example, a dolphin named Akeakamai was trained to vocally imitate computer generated sounds with a high degree of accuracy [18]. With two of the model sounds, the dolphin produced a faithful imitation but a full octave above and below the model sound, in what appears to be a case of octave generalization. In another study, a dolphin named Phoenix demonstrated the ability to classify sequences of tones as either having an ascending or descending sequence [19]. She was later able to generalize this ability to novel sequences a full octave above her training stimuli [19]. This ability, however, appears to have been slowly learned over the course of four experiments after conducting thousands of trials. During the initial transfer tests in experiment I, Phoenix required hundreds of trials over multiple sessions to reach the discrimination criteria of 80% correct and the authors concluded that the dolphin Phoenix “did not possess a robust concept of frequency contour” during that stage of the study (Ralston & Herman, 1995; p271). The study suggests that recognition of frequency-shifted tone sequences (octave generalization) with the dolphin Phoenix was not spontaneous and was the culmination of substantial long-term training.

Humans easily recognize melodies that have been frequency transposed [20], played with different instruments, and played at different speeds. Children easily learn to sing songs though imitation, but often produce songs within a preferred frequency range that does not match the exact pitch of the original model [21]. Octave generalization in humans is well established, but is rare in non-human species. For example, starlings (*Sturnus vulgaris*), cowbirds (*Molothrus ater*), and mockingbirds (*Mimus polyglottos*), all produce complex frequency-modulated (FM) songs [22]. However, all of these birds failed to recognize simple frequency contours that were frequency transposed outside of their training range [22]. The recognition system of these birds appears to be highly constrained by absolute pitch [23].

The question remains, how robust is the dolphin recognition system for FM whistles that may vary in frequency, duration, and amplitude while retaining consistent frequency contours? Does recognition of acoustically transformed sounds require substantial training like the dolphin Phoenix or is their ability to recognize acoustically transformed sounds spontaneous? To answer some of these questions a dolphin was presented with a three-alternative, matching-to-sample (MTS) task. The dolphin learned to associate three FM baseline sounds with three objects, then was tested with sounds that were transformed by amplitude, duration, or frequency transposition while still preserving the frequency contour of each baseline sound. The dolphin Phoenix’s ability to recognition frequency transposed tone sequences [19] predicts dolphins may be able to similarly recognize frequency transposed whistles. The same MTS task using the same stimuli, and non-verbal training and testing procedure were presented to a group of human participants for comparison purposes. Analysis of errors between the dolphin and humans along with post-experimental interviews with the human participants can illuminate possible processing mechanisms and decision strategies the dolphin may have been employing. Similar human-dolphin comparisons have been made in echolocation discrimination tasks, and have provided valuable insight into the acoustic cues dolphins may have utilized [24–26].

## Materials and Methods

### Participants

An Atlantic bottlenose dolphin (SAY, female, age 36, *Tursiops truncatus*) with extensive experience in cognitive [27, 28] and psychophysical testing [29, 30] participated in the experiment. She had normal hearing sensitivity at the frequencies tested as determined via auditory evoked potential testing [31]. She was housed in 9 m × 9 m or 9 m × 18 m floating netted enclosures (pens) located in San Diego Bay, California. Prior to this experiment SAY had participated in an auditory masking experiment with the baseline sounds (see below) utilizing the same procedures as the current experiment. The study followed a protocol approved by the Institutional Animal Care and Use Committee of the Biosciences Division, Space and Naval Warfare Systems Center Pacific, and all applicable U.S. Department of Defense guidelines for the care of laboratory animals.

Thirty human participants (20 males and 10 females) volunteered to participate in the study. Participants ranged in age from 19 to 34 years (*M* = 21.8). All participants were students at the Rochester Institute of Technology or residents of Rochester, NY. All of the participants were tested for normal hearing using a hearing test spanning 250–8,000 Hz (Digital Recordings, 2014). No participants reported any hearing difficulties and no participants showed any signs of hearing loss in the hearing test. Participants also filled out a questionnaire about their musical experience and ability level, and participants reported 0–18 years of musical experience (*M* = 4.9 years) and averaged 2.8 in ability level on a scale from 1–7. Participants who completed the study were given course credit for a psychology class or received $10. This study was approved by the Rochester Institute of Technology Institutional Review Board. Participants provided written informed consent before beginning the experiment.

### Stimuli and procedure

#### Dolphin acoustic stimuli

**Fig 2:**
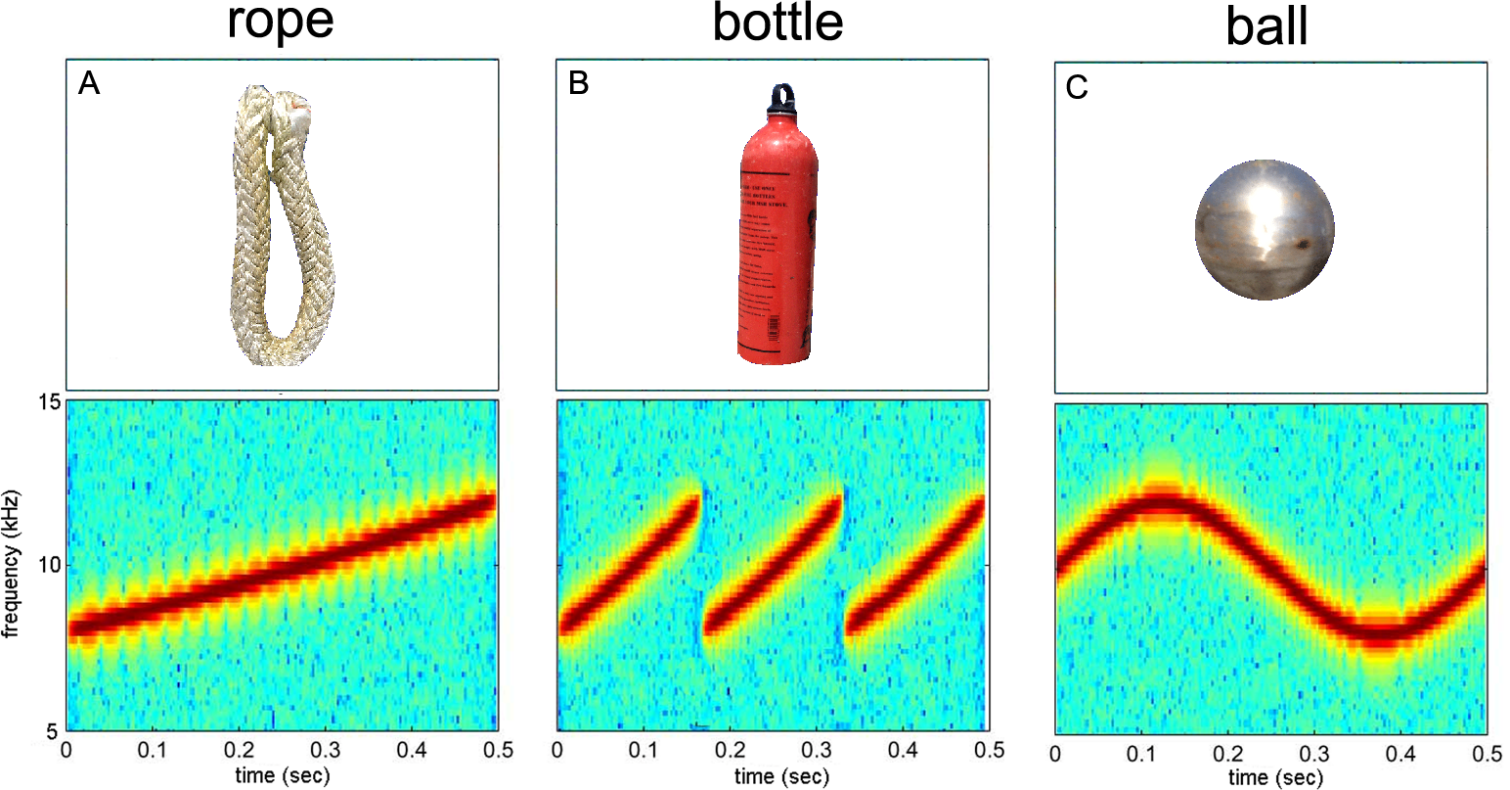
Baseline objects and their associated sounds. (A) “Rope” was a linear upsweep frequency contour. (B) “bottle” was a repeating linear upsweep, while (C) “ball” was a single cycle of a sinusoid. All baseline sounds were 500 ms in duration with equal bandwidths between 8 kHz and 12 kHz, with a center frequency of 10 kHz.

The current study was leveraged off of a previous auditory masking study (not reported here) which used the same “baseline” FM tones and a similar experimental paradigm. The same FM tones were used in the current study, instead of actual recorded signature whistles, to avoid the substantial time requirement involved with retraining the dolphin with novel sounds (see Dolphin Training below). The three FM sounds were created to loosely mimic dolphin whistles (Fig 2). One sound, designated as “rope” was a linear upsweep frequency contour. A second sound, designated “bottle” was a repeating linear upsweep, while “ball” had a frequency contour that was a single cycle of a sinusoid. All sounds were designed to be identical except for the frequency contour which is used for recognition. All baseline sounds were 500 ms in duration with equal bandwidths between 8 kHz and 12 kHz, with a center frequency of 10 kHz. The baseline sounds were presented at a sound pressure level (SPL) of 130 dB re: 1 μPa.

**Fig 3:**
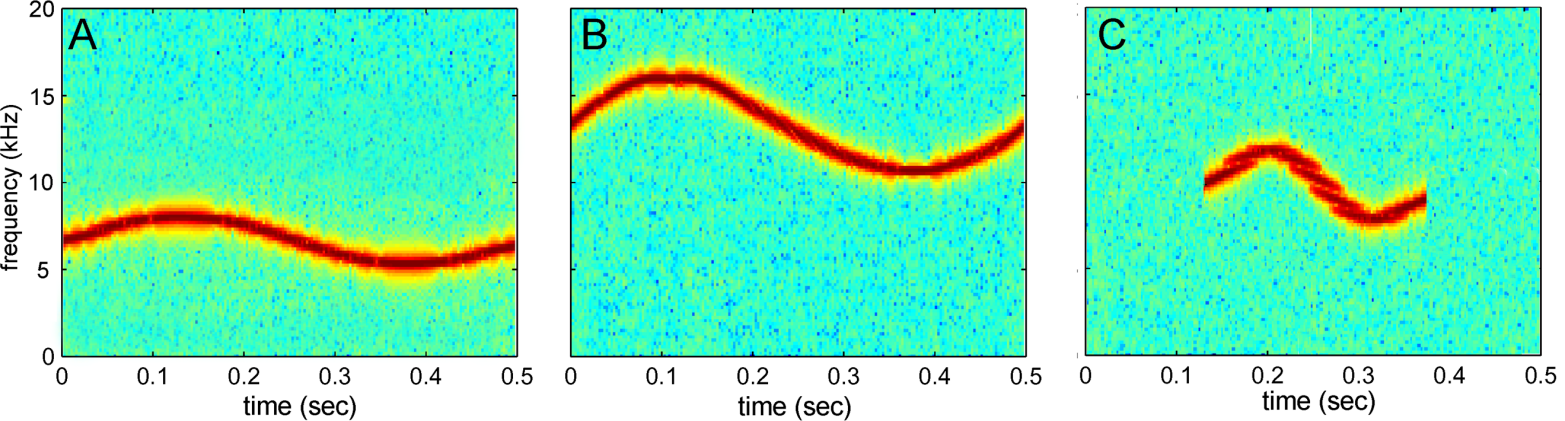
Examples of transformed “ball” test sounds. (A) ball has been transposed ½ octave down (re: baseline stimulus) in frequency, (B) shifted ½ octave up in frequency, and (C) the duration has been halved to 250 ms.

Test sounds were composed of acoustic modifications to the baseline sounds in SPL, center frequency, or duration. However, the frequency contours of the sounds were preserved (see Fig 3). SPL varied between 120 dB and 140 dB (re: 1 μPa) in 5 dB increments, for a total of five levels. Dolphins often produce whistles with source levels of approximately 160 dB re 1 μPa [7, 32]. The 130 dB baseline level represents the received level that a dolphin might hear if it were approximately 30 m from a whistling conspecific (assuming spherical transmission loss). For frequency modifications, sound were frequency transposed rather than frequency shifted. Frequency transposition is a multiplicative transformation and preserves the ratio (Q) of center frequency (CF) to bandwidth (BW) where Q = cf / BW. Frequency shifting is additive and preserves absolute bandwidth. Frequency transposition is more appropriate than frequency shifting because the frequency resolution of the dolphin auditory system has constant Q-qualities at the frequencies tested [33]. Center frequencies were adjusted to ½ octaves above and below the 10 kHz baseline sounds, as well as 1 kHz above and below the baseline sounds, for a total of five different sounds. Test sound durations were presented at 250, 400, 500, 600, and 1000 ms, for a total of five different sounds. Alterations to baseline sounds occurred to only one acoustic dimension (e.g., SPL, frequency, duration) at a time. For example, a test sound never included a concurrent change in duration and SPL. Tests sounds consisted of a total of 45 different sounds (Table 1).

**Table 1:** Test sounds used in the dolphin experiment. There were three sound types (rope, bottle and ball). Each sound type had three sound dimensions (amplitude, frequency and duration). And each sound dimension had five different dimension levels that were tested, for a total of 45 different test sounds. All underwater dB levels had a reference level of 1 μPa. Frequency values reflect the center frequency of each sound.

Sound dimension	Dimension Level
amplitude (dB)	(dB re 1 μPa) 120, 125, 130, 135, 140
frequency (kHz)	(kHz) 7.07, 9.00, 10.00, 11.00, 14.14
duration (ms)	(ms) 250, 400, 500, 600, 1000

Each sound was a digitized, (16-bit Microsoft.wav file with a sampling rate of 192 kHz). All sounds had 50 ms onset-offset ramps to reduce spectral splatter. Each wave file was converted to analogue [National Instruments USB-6251 (Austin TX)], filtered [100 Hz–100 kHz, Krohn-Hite 3C series module (Brockton, MA)] attenuated [PA5, Tucker Davis Technologies (Alachua, FL)], amplified [Hafler P7000 (Tempe, AZ)], and projected into the water [ITC 1001 piezoelectric transducer [International Transducer Corporation (Santa Barbara, CA)]. The ITC 1001 projector had a resonance frequency of 16.5 kHz, with a transmission voltage response (TVR) of 149 dB re: 1 μPa / V @ 1 m. A correction filter (FFT filter, Cool Edit Pro 2.0) was applied to all stimuli to compensate for the TVR of the projector, to produce flat spectrum stimuli. Stimulus generation was controlled with a custom Labview program. Stimulus levels were calibrated to 130 dB (re: 1 μPa) before each session by measuring the signal at the position that would be between the dolphin’s lower jaw (mandibular windows) during the experiment. Sound pressure levels were measured with a B&K 8105 (Copenhagen, Denmark, amplified [Reson VP1000 (Slangerup, Denmark)], filtered [100 Hz– 100 kHz, Krohn Hite 3C series module (Brockton, MA)], and digitized [606.1 kHz update rate, 16-bit resolution; National Instruments, USB-6251]. Signals were again measured after each experimental session and if the difference between pre-calibrations and post-calibrations was greater than 4 dB, the session’s data was rejected from analysis. This never occurred. When the SPL of the sounds was an independent variable, SPL was adjusted by a manual attenuator [PA5, Tucker Davis Technologies (Alachua, FL)].

#### Human acoustic stimuli

The same baseline sounds and test sounds used with the dolphin subject were presented to the human participants (Table 2). However, the sounds were down-sampled (44.1 kHz) and frequency transposed (preserving the sounds’ Q ratios) to lower frequencies (center frequency of baseline sounds = 1000 Hz) to facilitate the human range of sensitivity. Amplitude adjustments were made by changing the output voltage using Audacity 2.0.5 (Audacity, 2013). Sound pressure levels were calibrated using a calibrated digital sound level meter (Extech Instruments Model JTS-1357, 2012). Baseline sounds were presented at 70 dB (re: 20 μPa) and test sounds varied between 60 and 80 dB in 5dB steps. Instead of being presented with the physical objects (ball, bottle, rope), human participants viewed color photographs of each object printed on white paper (21.59 cm x 27.94 cm).

**Table 2:** Test sounds used in the human experiment. Each sound type had three sound dimensions (amplitude, frequency and duration). And each sound dimension had five different dimension levels that were tested, for a total of 45 different test sounds. All in-air dB levels had a reference level of 20 μPa.

Sound dimension	Dimension Level
amplitude (dB)	(dB re 20 μPa) 60, 65, 70, 75, 80
frequency (kHz)	(Hz) 707, 900, 1000, 1100, 1414
duration (ms)	(ms) 250, 400, 500, 600, 1000

#### Dolphin Training

**Fig 4:**
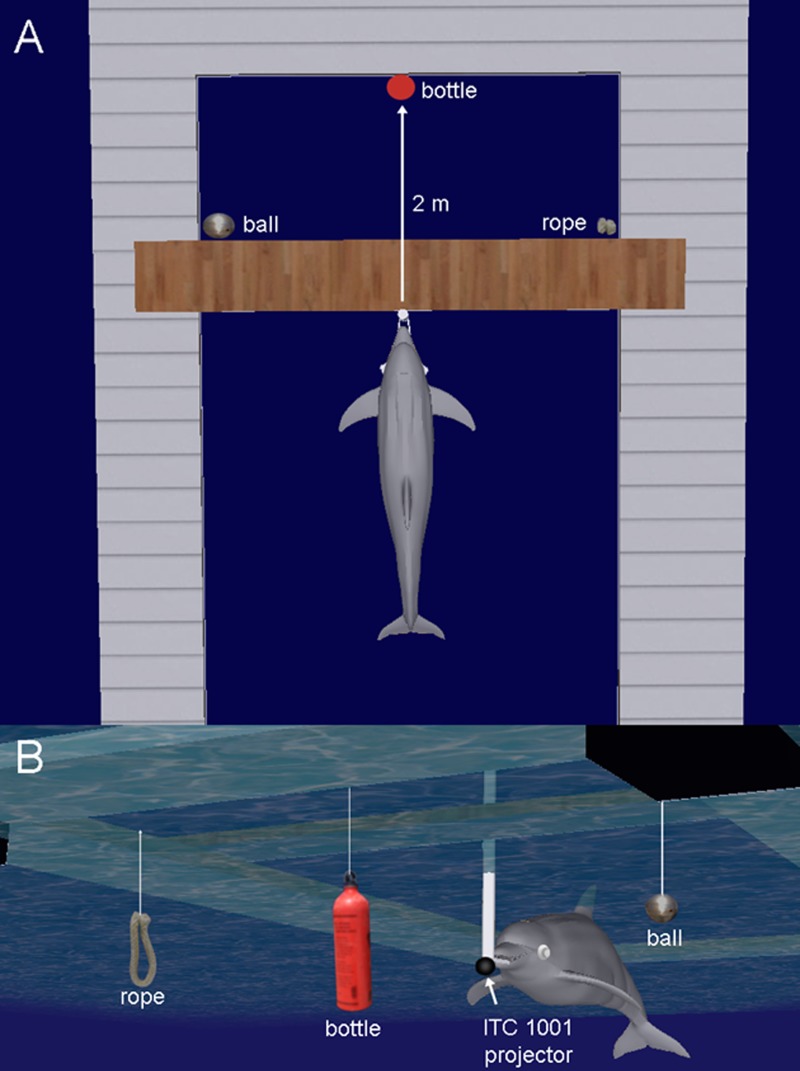
Experimental pen and objects. (A) Above view and (B) underwater view. During each trial, the dolphin stationed on an underwater biteplate at a 2 m radial distance from a nylon rope, a water filled aluminum bottle and an air filled steel sphere. Each of the three objects was suspended by monofilament at the same depth as the bite plate. The locations of the objects never changed.

The dolphin was trained to perform a three-alternative forced choice, matching-to-sample task. During initial training, the dolphin was required to station on an underwater biteplate (Fig 4). A single object, the nylon rope, was positioned at a fixed distance of 2 m from the biteplate stationing device, and at the same depth as the biteplate (approximately 50 cm below the surface). The depth of the biteplate was not arbitrary, but provided the dolphin with contextual information about the tasks she was required to perform. Other biteplates (e.g., a 2 m depth biteplate) and stationing devices (e.g., a stationing hoop) are used for different tasks including auditory detection tasks and echolocation discrimination tasks. No other objects (bottle or ball) were presented. Upon presentation of the rope sound (130 dB) the dolphin was immediately cued with a light *splash* on the water surface (near the rope) to direct the dolphin’s attention to the nylon rope and then swim and touch the rope with her rostrum. When the dolphin correctly touched the rope in response to the “rope” sound, a buzzer “bridge” sound was projected into the water informing the dolphin that she was correct at which point she returned to her trainer to receive fish reinforcement. After several cued training trials, the *splash* cues were gradually faded out from the procedure until the dolphin was reliably responding to the rope sound without being cued. “Reliably responding” was operationally defined as at least 80% correct for 10 consecutive trials. The procedure was then repeated for the bottle sound and the bottle object until the dolphin was reliably responding without a cue. At this point, both the rope and bottle objects were simultaneously presented and the presentation of the rope sound or bottle sound was randomized during each trial. The rope and bottle always remained at the same locations (Fig 4). If the dolphin swam and touched the correct object (e.g., touched the bottle in response to the bottle sound) she received the buzzer bridge followed by fish reinforcement. If she touched the wrong object (e.g., touched rope in response to the bottle sound) she received a “splash” call back which indicated an incorrect choice and instructed her to return to the trainer’s station where she did not receive fish reinforcement. When she was reliably responding to both the rope and bottle sounds, the ball object and ball sound were introduced (simultaneously with the previous objects) and the new sound-object association was taught using the principle of exclusion [34]. Each training session typically lasted between 20 and 45 minutes and was typically conducted once or twice a day, three to five days a week.

Upon completion of training, the dolphin SAY participated in a series of auditory masking experiments (not reported here) which used the baseline sounds and the above trained paradigm. During the masking study, the dolphin SAY was never exposed to time or frequency transformed sounds. She was, however, presented with the baseline sounds at variable levels, with a variety of background noise (See Branstetter et al., 2013, for a description of the noise types). Between 2011-August and 2012-April, 109 days of formal training were conducted. One to three sessions were typically conducted during each training day depending on several factors including SAY’s participation in other research projects, participation in enrichment programs, health assessments, and willingness to participate in general. Each session typically was composed of 35 trials, but varied occasionally at the discretion of the training staff. Data collection from the auditory masking study occurred between May 2012 and September 2012. Between September 2012 and January 2013, short “maintenance” sessions (10–20 minutes) were conducted, approximately one to three times a week to ensure the dolphin did not forget the experimental procedure. The current experiment was conducted between January 2013 and March 2013.

#### Human Training

The training procedure for human participants was designed to mirror the non-verbal training procedure used for the dolphin participant. The only verbal instructions given to the participants oriented them to the general scope and length of the task without indicating the specific object-sound pairs. The participants had to discover the correct answers through trial and error during the training phase, just like the dolphin. The instructions serve to orient the participants to the basic procedure of a match-to-sample task. The dolphin had already participated in matching-to-sample tasks prior to the current study. The exact instructions stated to the participants were as follows: “You will be participating in a non-verbal sound-to-picture matching task. This is the same sort of task that has been previously completed by a dolphin subject. I can only communicate to you using non-verbal instructions (for example, arm gestures or pointing) except for the feedback I give you after you make a choice. To indicate your answer for each trial, you should point to a picture. You will receive verbal feedback (e.g., “correct/incorrect”) after you make a choice. Once the session begins, I cannot answer any questions and you should not use any verbal communication. I will tell you when we are halfway through the testing phase. As a reminder, the experiment will last around an hour and a half. Do you have any questions?”

In a typical training trial, a stimulus was played and then the participant touched one of the three object photographs to make a response. The experimenter provided verbal feedback (“correct” or “incorrect”) and then immediately began the next trial. In the first part of the training phase, only the rope photograph was placed in front of the participant. If the participant did not immediately touch the photograph of the rope after hearing the first baseline rope sound, the experimenter would either point to the rope photograph or grasp the participant’s hand and bring it to the photograph. The experimenter cued the participant to touch the rope by pointing at the photograph for three trials. Then the participant completed 10 uncued trials where the rope baseline sound was played and the experimenter did not point at the rope photograph. All trials for the rest of the training phase were uncued.

In the second phase of training, the participant was presented with the photograph of the bottle (the rope photograph was removed). Participants heard the baseline bottle sound for ten trials and had to point to the bottle photograph. The participant was then presented with both the rope and bottle photographs, and completed ten trials (five trials of each object presented in a random order). Participants had to achieve 90% (9 out of 10 correct) before moving on to the next phase. All participants achieved a perfect score. In the final phase of training, participant were presented with all three objects. The baseline ball sound was trained by the principle of exclusion. The final phase of training consisted of 15 trials (five trials of each object presented in a random order), where the participants had to achieve 90% correct to move on to the test phase. All participants met the criterion (*M* = 99.11%) Training took 2–4 min (*M* = 2.93 min) to complete.

#### Dolphin testing procedure and analysis

During each trial, the dolphin stationed on an underwater biteplate (Fig 4) and waited for one of three sounds (sample) to be presented (Fig 2). After the sound was presented, the dolphin’s task was to swim and touch one of the three objects (with its rostrum) which was uniquely associated with the sound (choice). For example, if the “rope” sound was presented (Fig 2A), the dolphin was required to swim and touch the rope. If the “bottle” sound was presented, the dolphin was required to swim and touch the bottle, etc. The three objects were a nylon rope (length = 0.5 m, diameter = 5 cm), a water filled aluminum bottle (length = 15 cm, diameter = 6 cm), and an air filled steel sphere (diameter = 8 cm). Each of the three objects was at a 2 m radial distance from the bite plate and suspended by monofilament at the same depth as the bite plate. The locations of the objects never changed and as a result, the dolphin could have learned an object association with the sounds or place association (physical location) with the sounds. During each trial, the dolphin trainer served as a blind observer, in that she had no knowledge of the underwater sound being presented but verbally communicated the dolphin’s response to a research staff member located in an adjacent equipment shack. If the dolphin touched the correct object, the research staff member (who had knowledge of the sound being presented) instructed the trainer to “bridge” the animal at which point, the dolphin received a buzzer sound (secondary reinforcer) which cued the dolphin to return to the trainer’s station and receive a single capelin for reinforcement. If the dolphin chose an incorrect object, the researcher instructed the trainer to “call back” the dolphin at which point a light hand-splash on the water surface gave the dolphin feedback of an incorrect choice and cued her to return to the trainer’s station. Incorrect responses resulted in no fish reinforcement. During each trial, the dolphin wore latex rubber suction cups on her eyes (a.k.a., eye cups) to prevent her from using vision. Thus, she used echolocation to navigate, identify and choose each object. The primary use of the eye cups was not to force the dolphin to use echolocation, but to prevent her from attending to visual distractions that appeared to be a problem during the training phase of this experiment. Eye cups are routinely used in echolocation studies that have shown dolphins can discriminate and recognize objects, and can navigate through their environment without the use of vision [35–37].

One to three research sessions were conducted each workday. Each session was composed of ten warm up trials, fifteen test trials, and ten cool down trials. The warm-up and cool-down trials were composed of baseline sounds. The primary purpose of the baseline trials was to gauge the animal’s motivation prior to, and after the experimental trials by providing the dolphin with relatively “easy” trials. If her performance was less than 80% for the warm-up or cool-down trials, the data from the session were rejected. The cool-down trials also helped end each session on “a positive note,” which helped ensure that the dolphin would be motivated to participate in future sessions. Baseline sounds also aided in maintaining the dolphin’s performance. Sound types (i.e., rope, bottle, and ball) were presented in a pseudo random order on any given trial [38].

The fifteen test trials were composed of test sounds (Table 1), where each of the three sound types was tested five times each, in a pseudo random order. Only one sound dimension (i.e., amplitude, frequency, duration) was tested within a session, and the five dimension levels were presented in a pseudo random order. The sound dimensions were presented in an ABBA counterbalanced format (i.e., amplitude, frequency, duration, duration, frequency, and amplitude) to reduce any potential learning effects. Each of the 45 test sounds was tested ten times each for a total of 450 test trials.

Statistical analyses were conducted using R [39]. Polynomial regression was used to determine if acoustic dimension and sound type were related to proportion correct. Polynomial models were chosen instead of linear models because test sounds were both increases and decreases (in SPL, frequency, or duration) from the baseline sounds resulting in greater PC differences as levels of the acoustic dimensions departed from baseline values. This resulted in inverted “U” shaped proportion correct functions. Model simplification was performed using stepwise deletion to determine which predictor variables were significant.

#### Human testing procedure and analysis

A single experimenter tested the participants in a quiet room. The stimuli were presented to the participant and the experimenter via two sets of Bose on-ear headphones (Bose Corp, 2009) from a MacBook Pro laptop computer (Apple Inc., 2011). The sounds were played using QuickTime Player version 7.6.6 (Apple Inc., 2010). The participant sat facing the experimenter and the photographs, but was unable to see the computer screen. The participants were instructed that they would be completing a nonverbal sound-to-picture matching task before the training procedure began (see above). No further verbal instructions were given before the start of the test phase. The task was designed to be as similar as possible to the three-alternative forced choice match-to-sample task completed by the dolphin subject except that the participants completed an interview after the training and testing phases were finished.

The test phase began immediately after training was complete. There were 18 test sessions (6 sets with 3 sessions per set). Each set of 3 sessions contained only one type of acoustic transformation (amplitude, duration, or frequency). The order of the 18 sessions followed an ABBA counterbalanced design. Participants were randomly assigned to one of three orders. Order A followed the session order used for the dolphin subject (amplitude set, frequency set, two duration sets, frequency set, amplitude set). Order B began and ended with a duration set (duration set, amplitude set, two frequency sets, amplitude set, duration set) and Order C began and ended with a frequency set (frequency set, duration set, two amplitude sets, duration set, frequency set).

The session composition was exactly the same for the dolphin subject and human participants. Each session contained 35 trials. The first and last 10 trials of every test session were “warm-up” and “cool down” trials, in which the participant was presented with only baseline sounds. The middle 15 trials were the test trials, where all five levels of an acoustic transformation were presented once for each of the three sound types. The test phase consisted of 630 total trials (180 warm-up trials, 270 test trials, and 180 cool down trials). Each set of three sessions typically took 5–9 min (*M* = 6.32 min) to complete, and the entire test phase took 33–50 min (*M* = 40.23 min).

A short interview was conducted after the test phase was complete. Participants were supplied with a list of sound vocabulary terms that could be used to describe the simulated whistle sounds. The following operational definitions were provided: loudness (how loud or soft the sound is overall, related to the sound’s amplitude or intensity), pitch (how high or low the sound is overall, related to the sound’s frequency), length (how long each sound is overall, also called duration), timbre (the property in musical tones that makes it possible to distinguish one instrument from another when pitch and loudness are held constant), and frequency contour (the profile in a sound’s frequency across time). The experimenter then demonstrated each sound vocabulary term with auditory examples. Loudness was demonstrated with sequence of three pure tone sounds where the tones increased in volume (pitch and timbre were held constant). Pitch was demonstrated with two pure tone sounds played by a piano at a constant volume (880 Hz vs. 220 Hz). Duration was demonstrated with three notes of different lengths (1 s, 2 s, 4 s) played by a grand piano (pitch and loudness held constant; generated in GarageBand version 5.1, Apple Inc., 2009). Timbre was demonstrated with pure tone sounds (middle C) of three different instruments (French horn, trumpet, and soprano saxophone; loudness held constant). Two different dolphin whistles were played to demonstrate different frequency contours (Discovery of Sound in the Sea, 2013). The sound vocabulary sheet displayed accompanying spectrograms that showed changes in frequency over time of the two dolphin whistles. Finally, the experiment played the baseline sounds of rope, bottle, and ball again for the participant, and named them before playing each sound. Participants were allowed to hear any of the three sounds as many times as they wished.

In the interview, participants were asked to describe the differences they heard between the baseline sounds using vocabulary terms, to describe the different cues they utilized to identify each of the three objects, and to try to identify the ways in which the baseline sounds were transformed during the test phase. The interview phase lasted 2–6 min (*M* = 4.03 min). The entire experiment took 54–76 minutes (*M* = 66.4 min) to complete.

## Results and Discussion

### Dolphin Performance

**Fig 5:**
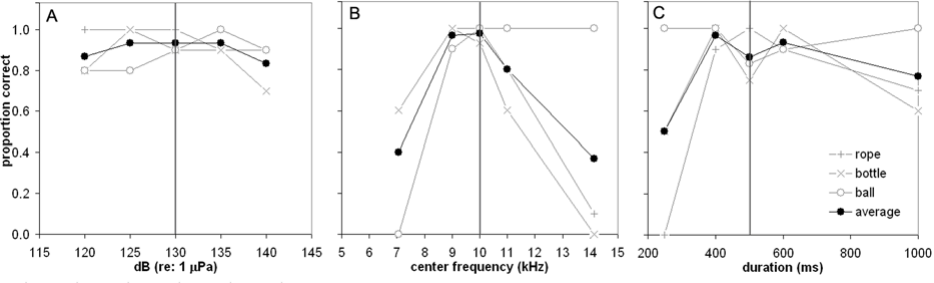
Dolphin Performance. Proportion correct as a function of (A) SPL (amplitude), (B) center frequency, and (C) sound duration. Each gray data point represents proportion correct for ten trials and black data points represent averages.

The proportion correct (PC) for warm-up and cool-down trials (a.k.a. baseline trials) was pooled together. Baseline PC was calculated for each sound type and each sound dimension (total of three PC measures for each sound type). Mean PC for each sound type in the baseline condition was 0.92, 0.96, and 0.96 for rope, bottle, and ball respectively. A one-way ANOVA resulted in no significant differences between these means, *F*(2,6) = 0.951, *p* = 0.438. For test trials, PC was calculated for each of the dimension levels, and is displayed in Fig 5. On average, a slight decrement in performance did occur at both 120 dB and 140 dB presentation levels. However, this decrement was not statistically significant, *F*(6,8) = 3.35, *p* = 0.059. SAY performed well regardless of loudness differences. Given that the SPL of whistles naturally vary at the sound source and due to range differences between the signaler and receiver, an amplitude invariant recognition system is not surprising.

A polynomial model which included frequency transposition, sound type, and an interaction between the two, was found to be a significant predictor of PC, *F* (8,10) = 26.76, *p* < 0.001. A modest reduction in PC occurred for CF = 11 kHz and a major reduction in recognition abilities occurs for both ½ octave shifts. In addition, an interesting pattern emerges in which the dolphin almost always chose ball when the center frequency was ½ octave higher than the baseline sounds (Fig 5). Both the rope and bottle sounds have a start frequency of 8 kHz while the ball has a start frequency of 10 kHz. If the dolphin weighted its decision on the start frequency (i.e., if high start frequency, choose ball) the erroneous defaulting to ball in the high frequency conditions can be explained. Of course, the “high-frequency start” rule cannot explain all of the errors the dolphin made and additional decision mechanisms are required. However, transpositional invariance does not appear to be a feature of this dolphin’s whistle recognition system.

Although duration had no significant effect on PC (*F*(6,8) = 1.45, *p* = 0.303), the pattern of errors is worth noting. Table 3 displays how many times the dolphin selected the wrong object during the test trials. Interestingly, when the duration of the rope sound was decreased to 250 ms, SAY erroneously chose bottle almost every time. Since the 250 ms rope sound is very similar to the first loop in the bottle baseline sound, the dolphin likely categorized the 250 ms rope as a single loop of the bottle sound.

**Table 3:** Errors made by the dolphin participant on each transformation type. The sound played is displayed in the left column of each matrix, and the dolphin’s choice is displayed along the top row. The sum column indicates the total incorrect trials for each sound type

			**AMPLITUDE**		
		**choice**			
		rope	bottle	ball	sum
**Sound**	rope		1	0	1
	bottle	1		2	3
	ball	0	5		5
					
			**FREQUENCY**		
			**choice**		
		rope	bottle	ball	sum
**sound**	rope		4	12	16
	bottle	1		14	15
	ball	4	7		11
					
			**DURATION**		
			**choice**		
		rope	bottle	ball	sum
**sound**	rope		14	1	15
	bottle	3		10	13
	ball	0	4		4

Table 4 displays immediacy of recognition with novel test sounds by examining the outcome of the trial the first time the new test sound was presented to the dolphin. Boxes with an “X” indicate a correct choice, while empty boxes indicate an incorrect choice. First-trial performance (a.k.a., transfer test) is important because there was no opportunity to learn the correct response due to feedback on previous trials. All trials after the first trial are subject to potential learning due to a positive or negative outcome. First trial performance was above chance for both SPL and duration but did not exceed chance performance for frequency transposition. The percent correct for first-trial performance was 91.6% for SPL (*p* < 0.001, cumulative binomial test, chance = 0.33), 50.0% for frequency (*p* = 0.063, cumulative binomial test, chance = 0.33), and 83.0% for duration (*p* < 0.001, cumulative binomial test, chance = 0.33).

**Table 4:** Immediacy of recognition by a dolphin of novel test sounds. An “X” indicates a correct choice the very first time the dolphin was presented with the stimulus, while an empty space indicates an incorrect choice for the first time.

SPL (dB)	120	125	135	140
Rope	**X**	**X**	**X**	**X**
Bottle	**X**	**X**		**X**
Ball	**X**	**X**	**X**	**X**
				
				
freq. (kHz)	7.07	9.00	11.00	14.14
Rope	**X**	**X**		
Bottle		**X**		
Ball		**X**	**X**	**X**
				
				
dur. (ms)	250	400	600	1000
Rope	** **	** X**	** X**	** **
Bottle	** X**	** X**	** X**	** X**
Ball	** X**	** X**	** X**	** X**

In summary, the dolphin’s recognition performance appears to be robust to transformation in amplitude and duration; however recognition did not generalize to the more frequency transposed sounds.

### Human Performance

**Fig 6:**
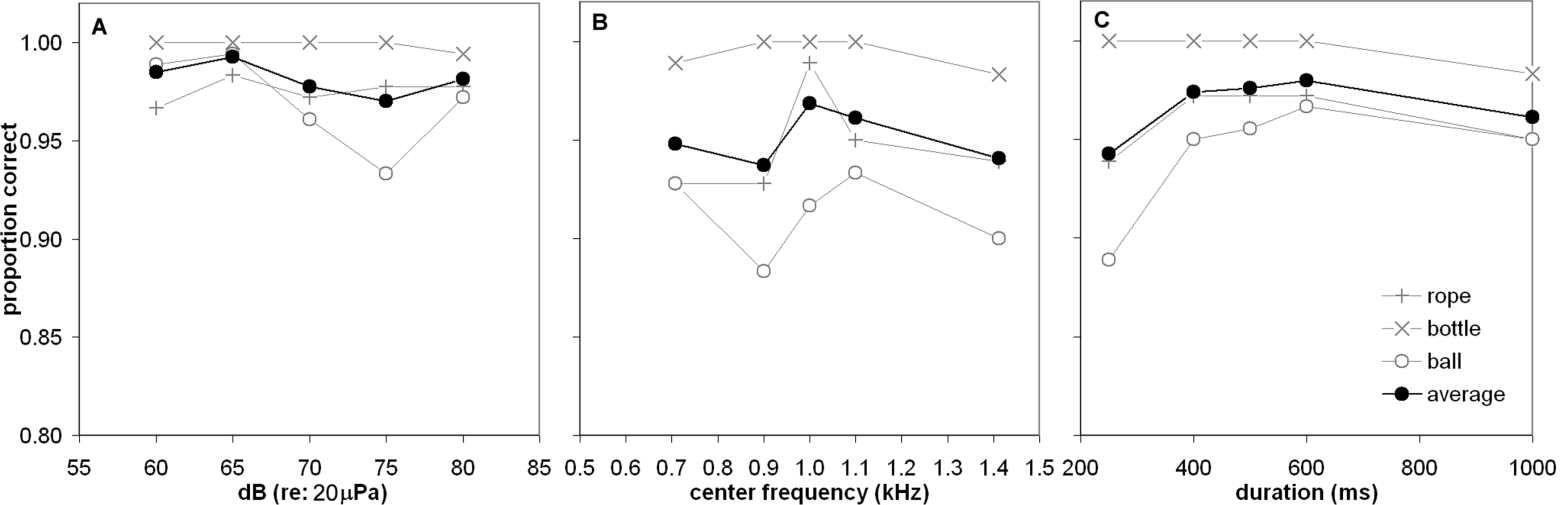
Human performance. Proportion correct as a function of (A) SPL, (B) center frequency, and (C) sound duration. Each gray data point represents proportion correct for ten trials and black data points represent averages. Note that the y-axis scale is different for Fig 5 and Fig 6.

The human participants were highly accurate at recognizing the sound types (Fig 6). Performance accuracy for warm-up and cool-down (baseline) trials during the test phase were pooled together and an average was calculated for each sound type. Mean performance accuracy in the baseline condition for each sound type was 97.8%, 99.8%, and 97.1% for rope, bottle, and ball, respectively. A 3 (group: order A, B, C) x 3 (sound type: rope, bottle, ball) analysis of variance (ANOVA) conducted on the warm-up and cool-down trials revealed a main effect of group; *F*(2, 27) = 3.36, *p* < 0.05; and a main effect of sound type; *F*(2, 54) = 7.89, *p* < .001. Performance on the bottle sound type was significantly better than on rope and ball sound types (Newman-Keuls, *p* < 0.05). Participants in order A (the same order given to the dolphin) performed significantly better than participants in order B (*M* = 99.8% vs. 97.0%) but there was no significant difference between orders C and B or orders A and C (C = 97.8%, Newman-Keuls, *p* < 0.05). It is unclear why this group effect occurred, but one possibility is that participants’ performance on baseline trials may have been influenced by their musical experience. Participants randomly assigned to group A averaged 7.5 years of musical experience, whereas participants randomly assigned to groups B and C averaged 2.5 and 4.6 years of experience, respectively.

Due to a nearly errorless performance on the bottle sound type on transformation trials (and lack of variance), it was excluded from all ANOVAs on the transformation test sessions. A 3 (group: order A, B, C) x 2 (sound type: rope, ball) x 5 (transformation level: 60 dB, 65 dB, 60 dB, 75 dB, 80 dB) ANOVA was completed on the amplitude test sessions with the last two factors as repeated measures. There were no main effects of group, *F*(2, 27) = 2.81, p = 0.08, sound type, *F*(1, 27) = 0.72, p = 0.40, or transformation level, *F*(4,108) = 2.33, p = 0.06. Like the dolphin, the human participants performed well regardless of transformations in amplitude.

A 3 (group: order A, B, C) x 2 (sound type: rope, ball) x 5 (transformation level: 7.07 kHz, 9.00 kHz, 10.00 kHz, 11.00 kHz, 14.14 kHz) ANOVA was completed on the frequency test sessions with the last two factors as repeated measures. There were no main effects of group, *F*(2, 27) = 2.7583, *p* = 0.08. There was a main effect of sound type, *F*(1, 27) = 10.56, *p* < 0.01, and an interaction effect between sound type and group, *F*(2, 27) = 3.47, *p* < 0.05. Post-hoc analyses revealed that participants in order C performed significantly better on the rope sound type (*M* = 92%) than on the ball sound type *(M* = 86%; Newman-Keuls, *p* < 0.05). Participants who began and ended the test with frequency sessions had a different pattern of performance than the other two groups. There were no main effects of transformation, *F*(4, 108) = 2.12, *p* = 0.08. Unlike the dolphin, there was no reduction in performance at any frequency transformation levels for the human participants.

A 3 (group: order A, B, C) x 2 (sound type: rope, ball) x 5 (transformation level: 250 ms, 400 ms, 500 ms, 600 ms, 1000 ms) ANOVA was completed on the duration test sessions with the last two factors as repeated measures. A main effect of group was found, *F*(2, 27) = 4.31, p = 0.02. Post-hoc analyses revealed that Group A (*M* = 99.8%) performed significantly better than Group B (*M* = 91.3%). There were no significant differences between Group A and Group C (*M* = 94.3%) or Group B and Group C (Newman-Keuls, p < 0.05). Participants who received the duration sessions after the other two transformation types (in the middle of the test phase) performed significantly better than those who received the duration sessions at the beginning and end of the test phase. A main effect of sound type was found, *F*(1, 27) = 4.64, *p* = 0.04. Post-hoc analyses found that performance on the rope sound type (*M* = 96.1%) was significantly better than performance on the ball sound type (*M* = 94.2%; Newman-Keuls, *p* < 0.05). Finally, a main effect of transformation was found, *F*(4, 108) = 4.19, *p* < 0.01. Post-hoc analyses revealed that performance on the 250 ms transformation (*M* = 91.4%) was significantly worse than the other transformation levels (400 ms: *M* = 96.1%, 500 ms: *M* = 96.4%, 600 ms: *M* = 96.9%, 1000 ms: *M* = 95.0%; Neumann-Keuls, p < 0.05). The drop in performance at 250 ms mirrors the dolphin’s performance.

**Fig 7:**
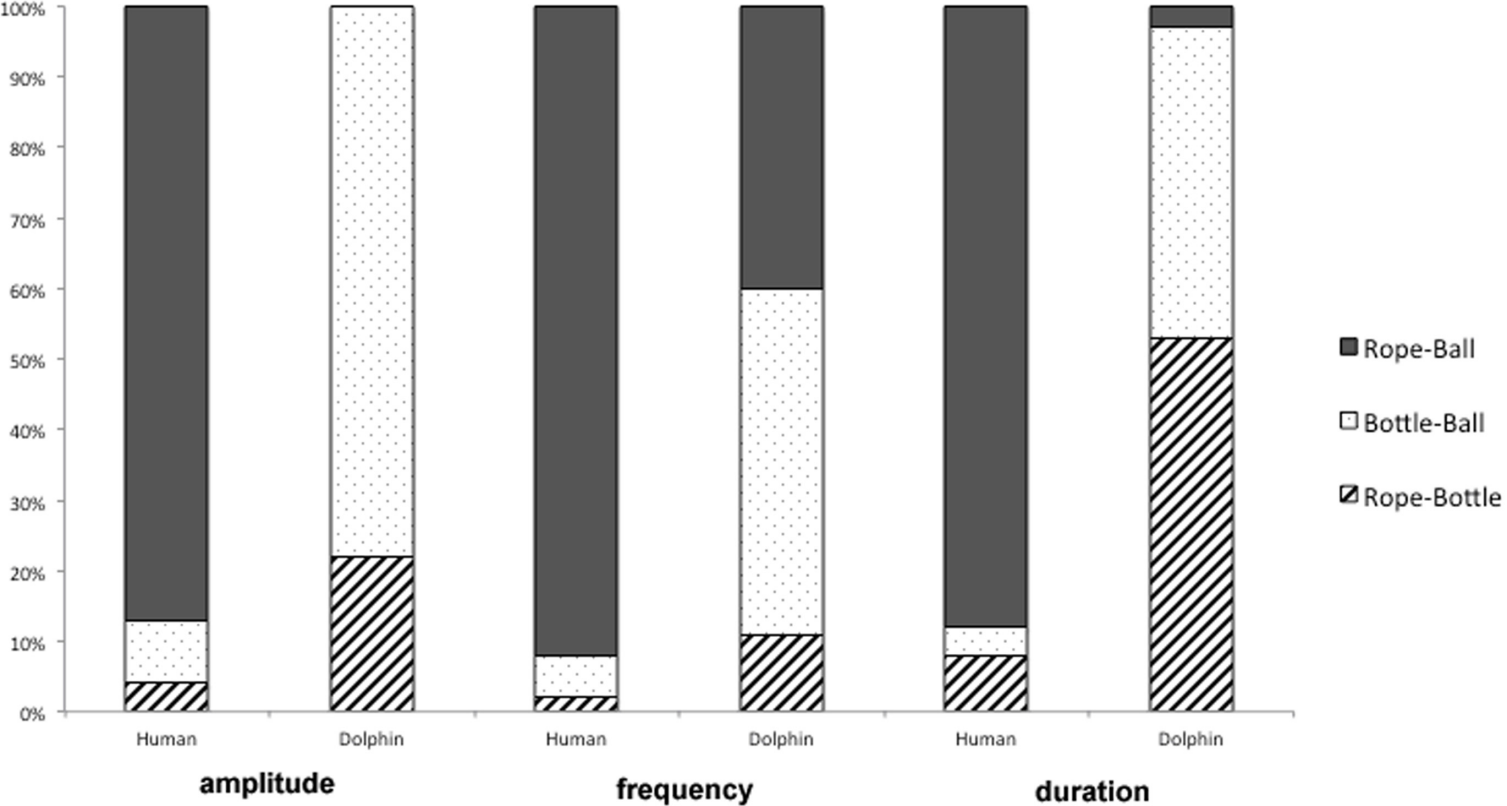
Comparison of errors (confusions between sound types) made by the human participants and the dolphin for each sound transformation.

The human participants made different errors than the dolphin subject (Table 5, Fig 7). The humans confused the rope and ball most often, whereas the dolphin confused the bottle and ball or rope and bottle more frequently. The human participants found the bottle sound type to be very distinctive (three short sounds) compared to the rope and ball sound types (each single sounds), and therefore almost never erred on bottle sounds.

**Table 5:** Errors made by human participants (*N* = 30) on each transformation type. The sound played is displayed in the left column of each matrix, and the participant’s choice is displayed along the top row. The sum column indicates the total incorrect trials for each sound type.

			**AMPLITUDE**		
		**choice**			
		rope	bottle	ball	sum
**sound**	rope		2	18	20
	bottle	0		1	1
	ball	23	3		26
					
			**FREQUENCY**		
			**choice**		
		rope	bottle	ball	sum
**sound**	rope		0	48	48
	bottle	1		3	4
	ball	76	5		81
					
			**DURATION**		
			**choice**		
		rope	bottle	ball	sum
**sound**	rope		3	30	33
	bottle	3		2	5
	ball	49	5		54

The errors made by human participants on amplitude transformations were primarily between rope and ball (41 errors, 87% of errors). A small proportion of errors were made between bottle and ball (4 errors, 9% of errors) and rope and bottle (2 errors, 4% of errors). The ball sound type was commonly mistaken for the rope sound type, accounting for 23 of the 41 errors, mostly occurring at the 75 dB transformation. In addition, the rope sound type was confused for the ball sound almost equally on all transformation levels, accounting for 18 total errors.

The most errors by human participants were made in the frequency sessions. A total of 133 errors were made, primarily between rope and ball (123 errors, 92%). A small proportion of errors were made between bottle and ball (8 errors, 6%) and rope and bottle (2 errors, 2%). The ball sound type was confused for the rope sound type most often across all transformation levels (including the baseline level), accounting for 76 of the 123 rope-ball errors. The rope sound type was confused for the ball sound type less often, but accounted for 48 of the 123 rope-ball errors. The errors were distributed nearly evenly across all transformation levels, except for the baseline level of 1000 Hz, where the rope sound type was confused for the ball sound type only twice.

A total of 91 errors were made in the duration sessions, primarily between rope and ball (79 errors, 87%). The same proportion of errors was made between bottle and ball and rope and bottle (6 errors or 7% each). The ball sound type was confused for the rope sound type most, accounting for 49 of the 91 errors, with the most errors (17) occurring in the 250 ms transformation. The rope sound type was confused for the ball sound type second-most often, accounting for 30 of the 91 errors. The majority of these errors occurred in the extreme transformations furthest from the baseline sound, 250 ms (7 errors) and 1000 ms (9 errors).

First trial performance for the human participants is shown in Table 6. The percentage of participants who answered the first trial correct for any given sound type and transformation ranged from 77% to 100%. In the conditions under which the fewest human participants answered the first trial correctly [ball at 900 Hz (77%), rope at 900 Hz (80%), ball at 400 ms (83%)], the dolphin was correct on the first trial in the analogous conditions. In the conditions under which the dolphin was incorrect on the first trial, a high percentage of the human participants answered correctly.

**Table 6:** Immediacy of recognition with novel test sounds for the human participants and the dolphin subject. Bold text indicates the dolphin was correct on her first trial, while regular text indicate the dolphin was incorrect on her first trial. The percent of human participants (N = 30) that achieved a correct answer on the first trial for each sound type and transformation is shown.

SPL (dB)	120	125	135	140
rope	**90**	**97**	**100**	**87**
bottle	**100**	**100**	100	**97**
ball	**100**	**97**	**87**	**100**
				
				
freq. (kHz)	7.07	9.00	11.00	14.14
rope	**90**	**80**	97	97
bottle	100	**100**	100	93
ball	90	**77**	**87**	**80**
				
				
dur. (ms)	250	400	600	1000
rope	93	**97**	**100**	93
bottle	**100**	**100**	**100**	**90**
ball	**87**	**83**	**90**	**93**

Twenty-nine of 30 participants reported hearing differences between the three baseline sound types. Of those 29 participants, all reported hearing differences in the frequency contours of the baseline sounds. The frequency of the ball sound type was mostly described to rise and fall in pitch. The rope sound type was described as a rising frequency contour, while the bottle sound type was described as 3 short rising pulses. Eighteen participants reported noticing differences in pitch in the baseline sounds. On average the rope was reported to have the highest pitch and the ball was reported to have the lowest pitch. Sixteen participants reported noticing differences in the duration of the baseline sounds. The ball was reported to have the longest length and the bottle was reported to have the shortest length. Six participants reported noticing differences in the amplitude levels of the baseline sounds. The bottle sound type was reported to be the loudest while the ball was reported to be the quietest. It is unclear whether the participants were really referring to the baseline sounds or the transformed sounds as all three baseline sounds were actually presented at the same loudness and the same duration.

Twenty-nine of 30 participants reported confusing the ball and rope sound types most often. Twenty-one participants thought the two sound types were similar in pitch, length, and timbre. Eighteen of the 29 thought they were similar in loudness, while 15 thought their frequency contours were similar. Most participants commented that the ball and rope sound types were similar because they were one continuous sound, whereas the bottle sound type was three short sounds. Even when different acoustic features of the sounds were transformed, the bottle sound type was still easily discriminable because it retained its pattern of three short burst sounds. In discriminating the three sound types, frequency contour was reported to be the most utilized cue (used by 28 participates for the ball sound type, 27 for the bottle sound type, and 26 for the rope sound type). Pitch was second most important in discriminating ball and rope (reported by 16 and 14 participants, respectively), but not as many reporting listening for pitch to discriminate bottle (only two participants). When asked what acoustic transformations were made to the baseline sounds (amplitude, duration, frequency), the majority of participants were able to name the three types (amplitude: 24 of 30, frequency: 25 of 30, duration: 26 of 30). Fifteen of the 30 also reported transformations in timbre, which participants may have confused for frequency transformations.

### General Discussion

In the current study, the dolphin’s ability to recognize frequency contours appears robust to changes in amplitude and duration, but constrained by absolute frequency. Human subjects, however, had superior recognition abilities with proportions correct well above 0.90 for all transformation types. For all animal listeners in general, changes in sound amplitude are constantly occurring due to changing distances between sound sources and listeners, environmental reverberation as well as natural SPL variation produced by an animal. It’s not surprising that both the dolphin and human listeners recognized the test sounds despite a 20 dB range in SPL. A common feature of almost any sensory system is robust recognition despite a large range in stimulus intensity.

For dolphins, individual variation in signature whistle duration is typically smaller than the variation in test sounds presented to SAY. For example, Esch et al. (2009) reported variations of about 30% of the mean duration. Despite the larger variation in duration for this experiment, SAY was able to recognize most of the whistles even when the duration was doubled or halved. An exception to this was that the 250 ms rope sound which was misclassified as bottle on 9/10 trials and ball on 1/10 trials. The rope sound and the bottle sound have identical frequency contours (linear upsweep from 8 kHz to 12 kHz), except the bottle has three repeating loops. The misclassification errors for rope suggest that the dolphin perceived the 250 ms rope sound as a single loop of the baseline bottle sound. If this is true, dolphins may perceive looped whistles as repeating units of discrete information rather than a single unit of information, which is consistent with previous findings [11]. Animal acoustic signals are often repeated to increase detection [40] to emphasize the information contained in the signal, to reduce signal ambiguity [41] or to advertise motivational or emotional state [13]. Humans, however, reported that the repeating pattern of the bottle sound was a very salient feature used for recognition. This suggests dolphins and humans may group sounds based on different temporal characteristics.

The primary difference between the dolphin and human performance was recognition of the frequency transposed sounds. Humans could readily recognize the frequency transposed sounds while the dolphin had great difficulty. For the dolphin, both recognition of rope and bottle sounds had similar PC patterns where performance was excellent for center frequencies (CF) of 10 kHz and 9 kHz, a slight decrease in performance for CF = 11 kHz, and considerable performance decrements for both positive and negative ½ octave shifts. These errors cannot be attributed to basic sensory limitations since dolphins have excellent frequency discrimination capabilities [42]. The hypothetical source of the dolphin’s difficulties could be related to one or more of the following: 1) lack of plasticity in the perceptual recognition system of the dolphin, 2) limited experience in recognizing frequency transposed sounds, or 3) the dolphin adopted a cognitive strategy with the baseline objects that did not generalize to the transfer stimuli. The first two hypotheses may share a causal relationship. For example, lack of experience with frequency transposed signals may have led to rigidity in the dolphin’s recognition system. For bottlenose dolphins in the wild (and the dolphin SAY prior to the experiment), there may be little pressure to learn to generalize recognition of signature whistles across large frequency ranges. The frequency contour of a signature whistle serves as a stable, reliable, long-term “signature,” and therefore, constraints on whistle variability must occur. Perhaps that is why signature whistles of wild dolphins appear to lack substantial variability in the frequency domain. For example, the standard deviations of frequency parameters for signature whistles (minimum and maximum frequencies) are typically low and about 10% of the mean values. [11]. The signature whistle of bottlenose dolphins are well defined at three months of age [43], and for females, will remain stable for decades with no evidence of change over time [44]. Males, however will form long-term alliances with other males, and individuals that are highly associated with each other will show subtle similarities between their signature whistles [45]. Bottlenose dolphins have excellent long-term memories [46, 47], maintain long-term associations with other conspecifics [48], and must remember long-term complex social interaction between individuals in their fission-fusion societies [49, 50]. The long-term stability of signature whistles, therefore not only provides a means for long-term recognition of individuals, but may potentially limit their ability to recognize frequency transposed sounds that are rare under natural conditions.

Humans, can understand speech and recognize melodies that have been frequency transposed [51]. This ability may be linked to the fact that children learn speech by imitating the speech of older adults, where the fundamental frequency of a model utterance can vary considerably from person to person. Furthermore, children’s utterances are typically much higher in frequency that an adult model, thus children typically do not have the option to mimic the frequency content of an adult utterance but must produce a frequency transposed version at the child’s restricted or preferred frequency range. The same principle does not apply to dolphins. Imitation of another dolphin’s signature whistle shows a considerably much smaller relative difference between the average frequency of the model and the copy [52]. In addition, humans produce vocalization that change during the course of aging with substantial modifications to a voice’s fundamental frequency from childhood to adolescents and into adulthood. As humans age, the fundamental frequency of their vocalizations will decrease due to the enlarging of their vocal apparatus [53]. Sexual dimorphism in humans also results in male fundamental frequencies that can be an octave lower than females [54]. Human speech variability may be a contributing factor to the flexibility seen in speech recognition which accommodates large variations in pitch, timbre, duration, and amplitude [51]. The fundamental frequency of most mammal vocalizations are also correlated with body mass [55]. Large animals produce sounds with low-frequency fundamentals, and vice versa. However, odontocete cetaceans are a notable exception, typically producing (and hearing) very high frequency sounds for their relatively large body mass. The long-term stability seen in the fundamental frequency of dolphin signature whistles during pre and post-sexual maturity, and regardless of age-related body mass, may be a by-product of the high frequency demands of echolocation. Both echolocation clicks and whistles are thought to be produced by adjusting the tension and morphology of tissues associated with the phonic lips, rather than resonance of nasal air cavities [56]. As a result, large differences in the size of an animal’s air cavities (related to the animal’s over all mass) have little effect on the fundamental frequency of their phonations. For example, killer whales, which are highly sexually dimorphic, show little difference in the fundamental frequencies of calls between males and females [56, 57]. However, differences do exists in the harmonic content of calls, which appears to be related to nasal air cavity volume. Tissues are also less affected by large changes in hydrostatic pressure compared to air cavity volumes. During deep dives, the fundamental frequency of whistles should remain stable. This is consistent with finding that depth had little effect on whistle frequency content of deep diving pilot whales [58]. Beluga whales whistling at depth, produced changes to the harmonic frequencies presumably related to compressed air cavity volume. However, little change occurred to fundamental frequencies produced by tissues associated with the phonic lips [59]. All evidence to date suggests whistle fundamental frequencies are quit robust to changes in animal size and hydrostatic pressure, again suggesting that signature whistles are a very stable acoustic signal. Although the harmonic structure of a phonation may change with nasal air cavity volume due to animal size or hydrostatic pressure, in bottlenose dolphins, the fundamental frequency of a signature whistle is used for recognition, not the harmonic structure or voice components [16]. Consequently, there may be little to no selective pressure to recognize frequency transposed whistles, when they may not naturally occur.

In addition to lack of variability in dolphin signature whistles, SAY’s extensive experience with the baseline sounds during training may have biased her decision strategy. SAY completed thousands of trials with the baseline sounds before the current experiment began. The extensive prior experience could have hypothetically trained her to attend to a narrow range of acoustic features for recognition and may explain some of her errors with respect to frequency transposed sounds. For example, the ball sound, was only misclassified (but 100% of the time) when the CF was shifted down ½ octave. Performance with the ball sound may be understood if the animal was employing a start frequency rule. For example, the start frequency for the baseline ball sound was 10 kHz while the start frequency of the baseline rope and bottle sounds were 8 kHz. If her mental rule was “choose ball if the start frequency of the sound is higher” she would be correct on the baseline trials. If she applied the same start frequency rule for the test trials, she would default to ball for sounds that were frequency transposed to higher frequencies, which is exactly what she did. When presented with rope and bottle sounds that were shifted up ½ octaves, she almost always defaulted to the ball (see Table 3). SAY’s performance in the frequency transposition condition is in contrast with the results from Ralston and Herman (1995). In this study, the dolphin Phoenix was able to discriminate between tone sequences that decreased in frequency compared to tone sequences that increased in frequency, even when the sounds were shifted a full octave from her training sounds. However, Phoenix underwent considerable training to first discriminate decreasing sequences from constant frequency sequences. It was only after three experiments, and hundreds if not thousands of trials, was Phoenix able to generalize the tasks to novel stimuli a full octave from her training stimuli. The extensive training with frequency transposed tonal sequences may have allowed her to develop, over considerable time, the abstract rule of “if descending sequence then whistle, else remain silent.” SAY on the other hand, received no incremental training with frequency transposed stimuli prior to presentation with the test sounds. Perhaps if the degree of frequency transposition were gradually increased over the course of hundreds of trials, as in the case of the dolphin Phoenix, SAY may have also learned the concept of frequency generalization.

In the current study, the dolphin SAY’s ability to recognize frequency modulated tones was robust to changes in amplitude and duration, but constrained by absolute frequency. Although the dolphin SAY had extensive experience in cognitive tasks [27, 28] and psychoacoustic tasks [30], this study, as well as all studies with a limited subject pool, should be interpreted with caution. Replication of this study (and similar laboratory studies) is difficult due to obstacles associated with housing and long-term training. A whistle recognition study could be conducted in the field with a larger number of dolphins, using a well-established playback methodology [3, 60], and frequency transposed signature whistles, to confirm or refute the results from the current study.
